# The Advances in Novel Delivery Strategies for Hirudin Against Cardiovascular Diseases

**DOI:** 10.3390/ph19020204

**Published:** 2026-01-25

**Authors:** Mengjing Li, Tianxiang Yue, Jia Li, Tianze Tao, Tshepo Nkwane, Lai Jiang, Ranxiao Zhuang, Fanzhu Li

**Affiliations:** 1 School of Pharmaceutical Sciences, Zhejiang Chinese Medical University, Hangzhou 310053, China; mjeinngg1009@163.com (M.L.); txyue@zcmu.edu.cn (T.Y.); 202521014011155@zcmu.edu.cn (J.L.); 202521014011172@zcmu.edu.cn (T.T.); laijiang.sps@zcmu.edu.cn (L.J.); 2 International Education College, Zhejiang Chinese Medical University, Hangzhou 310053, China; tsheponkwane1997@gmail.com; 3 Xixi Hospital of Hangzhou, Zhejiang Chinese Medical University, Hangzhou 310023, China

**Keywords:** hirudin, mechanism, against cardiovascular diseases, drug delivery strategies, clinical application challenges

## Abstract

The natural polypeptide drug hirudin, a direct thrombin inhibitor, exhibits potent anticoagulant, anti-myocardial fibrotic, and anti-inflammatory effects in the treatment of cardiovascular diseases (CVD), but its clinical application remains limited by its low bioavailability, insufficient targeting capability, and bleeding risk. In recent years, the development of nanotechnology has enabled peptide drug delivery systems to demonstrate substantial promise in medical practice. Significant progress has been made in overcoming limitations and enhancing therapeutic efficacy against CVD through the use of Hirudin-based drug delivery systems by addressing drug stability in vivo, improving targeting ability, and ultimately achieving responsive release. This paper systematically reviews the mechanisms of action, clinical applications, and novel delivery strategies of the peptide drug hirudin in the treatment of CVD, with a particular focus on recent advances in hirudin-based drug delivery systems, and it also looks forward to future research directions for hirudin delivery systems, including the development of scalable intelligent carriers, the construction of real-time feedback systems, and the establishment of standardized in vitro and in vivo evaluation systems, aiming to present novel strategies for safe and efficient treatment of CVD.

## 1. Introduction

Peptide drugs possess characteristics of high target affinity, selectivity, and low immunogenicity and are widely applied in the treatment of metabolic diseases, cancer, and cardiovascular diseases [1,2]. Among them, Hirudin, a natural polypeptide derived from the extract of the salivary glands of *Hirudo medicinalis* leeches, has been identified as a quintessential example in the field of cardiovascular disease treatment [3]. Modern scientific research on hirudin originated in the late 19th century. In 1884, British scientist Haycraft first identified a substance with anticoagulant properties in leech salivary glands; in 1904, British scientist Jacoby designated this compound ‘hirudin’ [4]. By the 1950s, advancements in peptide and protein separation technologies enabled researchers to successfully purify hirudin. In 1968, German scientist Markward summarized that hirudin possessed a 65-amino-acid polypeptide structure weighing approximately 7000 Daltons [5]. Hirudin, the most potent known direct thrombin inhibitor, demonstrates significant potential in the treatment of CVD; however, its clinical application still faces numerous challenges [6,7,8].

Although hirudin has prominent advantages, including high bioactivity, high specificity, and low toxicity [9], it still faces several challenges, including susceptibility to enzymatic degradation, a short half-life, and low bioavailability during clinical applications [10]. To overcome these defects, high-dose, high-frequency dosing regimens are used in clinical treatment; however, they not only significantly increase treatment costs but also may cause severe adverse reactions such as bleeding [3,11]. This dramatically limits hirudin’s application. Supported by advances in nanotechnology, researchers design novel nanocarriers, perform surface functionalization, and construct stimuli-responsive drug delivery systems. These strategies effectively protect polypeptide molecules from degradation in physiological microenvironments; prolong their circulation time; and achieve efficient, controlled targeted delivery and release. These approaches successfully address peptide drug delivery challenges while providing a novel therapeutic strategy for hirudin application in the treatment of CVD [12,13,14,15].

Unlike prior reviews focused primarily on hirudin’s anticoagulant properties, this review systematically examines the pharmacological mechanisms of the polypeptide drug hirudin in the treatment of CVD. Compared with previous reviews, this article explicitly addresses delivery strategies designed to overcome existing therapeutic bottlenecks, including maintaining biological activity, enhancing targeting efficiency, and achieving controlled drug release. Furthermore, this paper also proposes future research directions for hirudin in CVD treatment and provides critical references for clinical applications of hirudin and other polypeptide drugs.

## 2. Mechanisms of Action of Hirudin Against CVD

Hirudin, a powerful anticoagulant, performs a critical function in CVD treatment. It produces efficient anticoagulation by specifically inhibiting the activity of thrombin and altering hemorheological properties. Beyond this direct anticoagulant role, it exhibits critical cardioprotective effects by anti-myocardial fibrosis and anti-inflammatory effects, which provide unique clinical advantages for the treatment of CVD [6,16,17].

### 2.1. Antithrombotic

As a critical pathological basis for CVD, thrombosis may trigger severe clinical events, including acute coronary syndrome (ACS) and venous thromboembolism (VTE) [11,18,19]. The acceleration of population aging drives the persistent increase in thrombotic disease incidence, posing a major global health challenge. As the first line of defense against clinical CVD, anticoagulant drugs serve a critical function in routine therapy and perioperative management [20,21,22,23]. Hirudin, the most potent known natural thrombin inhibitor, has received FDA and EMA approval for the prevention of thrombosis [24,25].

The core anticoagulant mechanism of hirudin is to inhibit thrombin directly, efficiently, and specifically, thereby blocking the coagulation reaction and achieving precise regulation of thrombus formation [26,27,28]. It plays an irreplaceable role due to its unique targeted inhibition ability in anticoagulant therapy.

**Figure 1:**
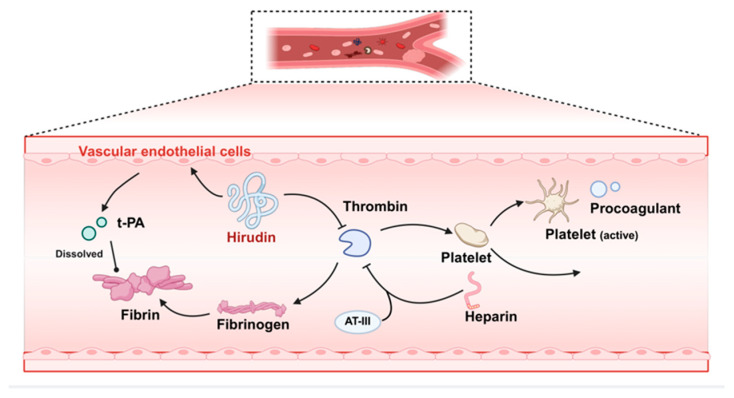
The anticoagulant mechanism of hirudin in CVD.

Hirudin forms a stable non-covalent complex with thrombin at a 1:1 stoichiometric ratio, inhibiting thrombin activity and blocking thrombin-catalyzed conversion of fibrinogen to fibrin, consequently inhibiting thrombus formation [6,29,30]. Unlike heparin, which requires antithrombin III (AT-III) as a cofactor, hirudin directly combines with thrombin independently of plasma AT-III levels, resulting in more stable anticoagulation. Furthermore, hirudin demonstrates multi-target antithrombotic ability by preventing thrombin through blockade of thrombin–platelet binding, thereby suppressing platelet activation and procoagulant mediators release, while stimulating endothelial-tissue-type plasminogen activator (t-PA) secretion to potentiate fibrinolysis (Figure 1) [7,31]. This dual mechanism is critical in post-thrombolysis anticoagulation therapy. When thrombolytic drugs recombinant-tissue-type plasminogen activator (rt-PA) exert effects, accompanying thrombin may cause secondary thrombosis. Hirudin prevents secondary thrombosis and reduces excessive t-PA consumption by continuously inhibiting thrombin activity, thereby maintaining fibrinolytic activity and lowering re-occlusion risk post-thrombolysis [32]. Therefore, hirudin is clinically combined with thrombolytics, such as rt-PA, to enhance thrombolytic efficiency and reduce the formation of secondary thrombosis [33,34,35].

### 2.2. Anti-Myocardial Fibrosis

**Figure 2:**
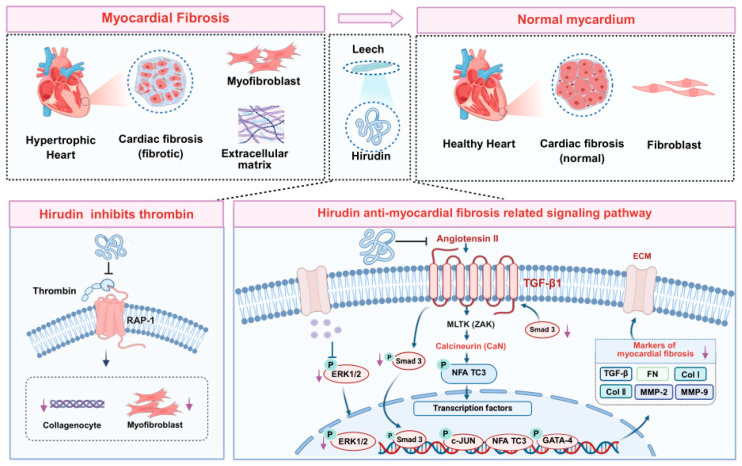
The mechanism of anti-fibrotic effects of hirudin in CVD.

Myocardial fibrosis is a critical pathological process in CVD, such as hypertension, myocardial infarction, and heart failure, which primarily manifests as the excessive activation of myocardial fibroblasts and abnormal deposition of extracellular matrix (ECM) (Figure 2) [36]. Research has shown that thrombin, as a promoter of fibroblast and collagen production, primarily exerts pro-fibrotic effects by activating protease-activated receptor 1 (PAR-1) and upregulating collagen mRNA. Hirudin can effectively block PAR-1 activation through specific thrombin inhibition, thereby reducing collagen production and suppressing fibroblast proliferation [37,38,39].

#### 2.2.1. Ca^2+^/CaN/NFATc Signaling Pathway

Calcineurin (CaN) is a key signaling mediator involved in inducing myocardial hypertrophy, and the CaN/NFAT pathway activated by Ca^2+^ overload is an important pathway in myocardial hypertrophy and fibrosis [40]. Angiotensin II (Ang II) demonstrates pro-fibrotic effects by upregulating angiotensin II receptor 1 (AT1R), CaN, NFATc3, and GATA4 through the Ca^2+^/CaN/NFATc signaling pathway [41]. In Ang II-treated H9c2 cells and spontaneously hypertensive rat (SHR) models, the expression of myocardial fibrosis markers, including transforming growth factor-β (TGF-β), matrix metalloproteinase-2 (MMP-2), matrix metalloproteinase-9 (MMP-9), type I collagen (COL-I), and type III collagen (COL-III) was upregulated alongside increased protein expressions of ZAK, p38, and JNK. Treatment with leech extract effectively reduced the expression of these pro-fibrotic factors [38].

#### 2.2.2. ERK1/2 Signaling Pathway

As a classical pathway within the mitogen-activated protein kinase (MAPK) family, the ERK1/2 signaling pathway plays an essential role in the process of myocardial fibrosis [42]. ERK1/2 activate NF-κB and AP-1 to stimulate the expression of important fibrosis marker matrix metalloproteinase-1 (MMP-1) [43]. The study illustrated that Ang II-induced cardiac fibroblast proliferation is mediated by the ERK1/2 pathway [44]. Hirudin downregulates levels of fibrosis-related factors, including TGF-β, MMP-2, MMP-9, COL-I, and COL-III, by inhibiting phosphorylation of ERK1/2; upregulating expression of the tissue inhibitor of metalloproteinase-2 (TIMP-2); and inhibiting MMP-2 activity and fibroblast proliferation/differentiation, thereby exerting anti-fibrotic effects [44].

### 2.3. Anti-Inflammatory

**Figure 3:**
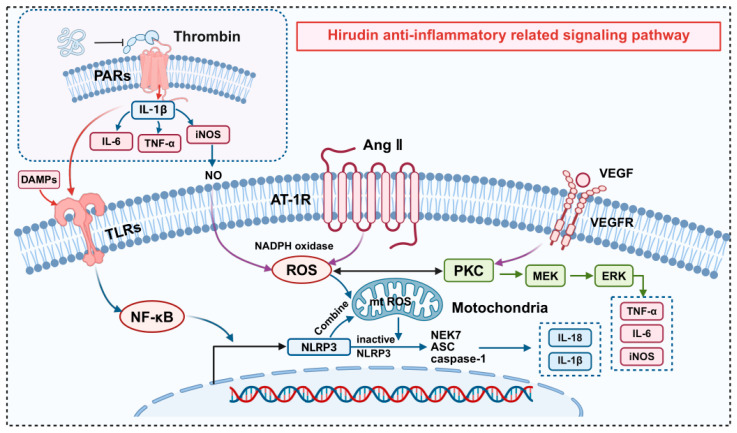
The mechanism of anti-inflammatory effects of hirudin in CVD.

Hirudin plays a significant anti-inflammatory role through a multi-target regulatory mechanism (Figure 3). It has unique value in the treatment of cardiovascular conditions associated with inflammation, including coronary heart disease, myocardial ischemia–reperfusion injury, and atherosclerosis [45,46].

Hirudin not only significantly reduces tissue damage but also effectively lowers local inflammatory reactions while enhancing tissue antioxidant capacity and protecting endothelial cells [47,48]. It primarily exerts effects by regulating NF-κB/NLRP3, PINK1/Parkin, EGF/VEGFR, and thrombin inhibition-related pathways. The synergistic actions of these multiple pathways make hirudin an ideal candidate for treating cardiovascular disorders related to inflammation.

#### 2.3.1. NF-κB/NLRP3 Signaling Pathway

Myocardial ischemia releases damage-associated molecular patterns (DAMPs) and pro-inflammatory factors (TNF-α, IL-1β), which activate the NF-κB signaling pathway by engaging toll-like receptors (TLRs), further inducing NLRP3 inflammasome activation through NLRP3, apoptosis-associated speck-like protein (ASC), and pro-caspase-1 oligomerization [49]. This process triggers the release of inflammatory factors, such as TNF-α, IL-1β, and caspase-1, leading to cell necrosis and expansion of the myocardial infarction area [50]. Recent studies demonstrate that hirudin exerts anti-inflammatory effects by regulating the DAMPs-TLRs-NF-κB axis to suppress NLRP3 transcription and cytokine production (TNF-α, IL-6) [51,52,53], while directly inhibiting NLRP3 inflammasome overexpression, thereby alleviating myocardial inflammation [45,54].

#### 2.3.2. PINK1/Parkin Signaling Pathway

Accumulated ROS damages mitochondria when the production and clearance of ROS are out of equilibrium, the ROS then bind to NLRP3, causing NLRP3 inflammasome activation and triggering inflammatory responses [55]. Studies on ROS-mitophagy-NLRP3 interactions reveal that hirudin activates the PINK1/Parkin pathway to promote mitochondrial autophagy (upregulating LC3-II/LC3-I ratio and BECN1), clear damaged mitochondria, inhibit NLRP3 inflammasome formation, and reduce inflammatory factors (IL-18, IL-1β). In Ang II-induced cardiomyocyte hypertrophy models, hirudin reduces ROS to ameliorate oxidative stress and exerts anti-inflammatory effects via the PINK1/Parkin pathway [53].

#### 2.3.3. VEGF/VEGFR Signaling Pathway

In chronic inflammation, the signaling pathway regulating angiogenesis and vascular permeability, comprising VEGF and VEGFR, is aberrantly activated. Thrombin induces NF-κB pathway activation by activating PAR-1, which promotes the release of IL-6 and TNF-α and inhibits the phosphorylation of VEGF/VEGFR-2, thereby inhibiting the Raf/MEK/ERK pathway [56]. Research shows that natural hirudin promotes angiogenesis in rat ischemic flap models by regulating p38, MAPK, and ERK expression [57].

#### 2.3.4. Anti-Thrombin-Related Signaling Pathways

Thrombin, a crucial modulator of inflammation and thrombosis, enhances protection against endothelial cell damage by activating proteinase activated receptors (PARs) and utilizing the PKC-ε/MAPK signaling pathway to induce decay accelerating factor (DAF) expression in human umbilical vein endothelial cells (HUVECs) [58]. Hirudin inhibits PAR-mediated monocyte/macrophage inflammatory factor (IL-1, IL-6, TNF-α) release and platelet activation, comprehensively blocking thrombin-mediated pro-inflammatory responses; it also directly inhibits thrombin to suppress the thrombin/PAR/p38/NF-κB signaling pathway and inflammatory responses [57].

## 3. Clinical Applications of Hirudin Against CVD

WHO data indicate that CVD rank among the foremost causes of global mortality [59]. Hirudin exhibits potent anticoagulant effects and is widely used clinically. However, natural hirudin, derived from the salivary glands of medicinal leeches, faces limitations, including limited production, high extraction costs, and difficulty in ensuring purity; in contrast, recombinant hirudin, as a gene-recombinant product using natural hirudin as a reference has lower production costs and is easier to scale up [60]. Natural hirudin can only be extracted from live leeches with extremely low yields. Recent advances in recombinant production utilizing microorganisms or algae offer a novel method for large-scale production, avoiding leech farming and complex extraction processes, significantly reducing costs, and breaking traditional animal extraction bottlenecks, thereby making recombinant hirudin production independent of leeches [61,62].

With the development of genetic engineering, recombinant hirudin and its derivatives have become a focus of research. Current clinical applications include recombinant hirudin (lepirudin and desirudin) and hirudin derivative bivalirudin (Table 1) [63,64,65,66,67]. These demonstrate excellent preventive/therapeutic effects for CVD, such as ACS, thromboembolism, and heparin-induced thrombocytopenia (HIT), exhibiting promising clinical prospects [8,9,68].

### 3.1. Acute Coronary Syndrome (ACS)

Acute coronary syndrome (ACS) encompasses a spectrum of clinical disorders resulting from a rapid decrease or interruption of coronary blood flow, classified as ST-segment elevation myocardial infarction (STEMI); non-ST-segment elevation myocardial infarction (NSTEMI); and unstable angina (UA) [68,69]. The underlying pathogenesis involves atherosclerotic plaque disruption, which initiates platelet accumulation and thrombus formation, producing partial or complete coronary artery occlusion and eventually causing myocardial ischemia or necrosis [11,68].

#### 3.1.1. Myocardial Infarction (MI)

Hirudin and its derivatives (e.g., bivalirudin) demonstrate a more favorable risk–benefit ratio compared with conventional heparin in anticoagulant therapy for patients with STEMI, particularly for high-bleeding-risk patients.

In the HORIZONS-AMI trial, Bivalirudin is considered a potent anticoagulant and safer drug in STEMI patients undergoing primary percutaneous coronary intervention (PCI) treated. Compared with the standard regimen of heparin combined with GP IIb/IIIa inhibitors, bivalirudin can significantly reduce major bleeding and results in fewer 30-day adverse clinical events [70]. This finding differs from the acute-phase treatment concept at that time, which centered on strengthening antithrombotic therapy and often came with a high risk of bleeding. It holds significant importance. This is also reflected in the latest data from previous large meta-analyses and the BRIGHT-4 trial. The GUSTO-IIb trial demonstrated that direct thrombin inhibitors (hirudin) further reduce the risk of death/myocardial infarction in ACS patients compared with heparin, exhibiting lower post-discontinuation reinfarction rates [71]. A meta-analysis of 35,970 ACS patients receiving direct thrombin inhibitors or heparin showed that hirudin/bivalirudin significantly reduced death/MI events and significant bleeding risks versus heparin [72]. The BRIGHT-4 trial confirmed that bivalirudin reduced bleeding risk by 52% [73].

The pharmacological basis for its excellent clinical performance lies in its unique mechanism of action, bivalirudin exerts antithrombotic effects by binding reversibly to the active site of thrombin and has a short half-life (about 25 min). This allows it to provide adequate anticoagulant effects during PCI procedures, while the drug’s effect rapidly diminishes postoperatively, which may help achieve a better balance between antithrombotic efficacy and bleeding risk. However, its clinical application is not without controversy. Early observations suggested it might increase the risk of acute stent thrombosis, prompting optimization of treatment regimens. Its efficacy in MI patients without thrombolytic therapy was evaluated in trials like ASIS-1 [74]. Nevertheless, this has further clarified the core challenge of modern anticoagulant therapy: how to achieve a dynamic balance between antithrombotic effects and bleeding risk. In summary, hirudin-like drugs represented by bivalirudin provide a new anticoagulant option for STEMI patients, especially those with a high risk of bleeding, through their unique pharmacological properties.

#### 3.1.2. Unstable Angina (UA)

Unstable angina (UA), an important type of ACS, has coronary atherosclerotic plaque rupture with thrombosis as its pathological basis. Against the background of this disease, the value of hirudin and its derivatives lies not only in their potent anticoagulant properties but, more importantly, in providing new tools and strategies for achieving precise antithrombotic therapy. Regarding treatment, studies show that recombinant hirudin optimizes individualized antithrombotic management during coronary angioplasty perioperatively through real-time plasma drug monitoring, thereby improving anticoagulation precision [74]. Bivalirudin is commonly used in percutaneous coronary intervention due to its short half-life (approximately 25 min) and predictable anticoagulant effect, especially suitable for patients at high risk of bleeding. This strategy can maximize the avoidance of ischemic or bleeding risks caused by insufficient or excessive dosage while ensuring antithrombotic efficacy, making it particularly suitable for high-risk patients with complex and variable coagulation function.

In terms of combination therapy, The TIMI study (randomized double-blind trial) confirmed that combining antithrombin agents (bivalirudin) with aspirin synergistically inhibits thrombus formation in UA. Results demonstrated that bivalirudin played a crucial role in early invasive therapy for ACS [75]. Aspirin inhibits the upstream of platelet activation, while bivalirudin precisely inhibits thrombin, which is involved in platelet aggregation and fibrin formation. The combination of the two is more direct and comprehensive in their mechanisms compared with traditional heparin. Therefore, the key role of bivalirudin in the early invasive treatment of ACS stems from its ability to provide potent and controllable anticoagulation without significantly increasing perioperative bleeding burden.

These data indicate that hirudin and its derivatives (bivalirudin) exhibit superior efficacy and safety compared with heparin in the treatment of UA and enables clinicians to prescribe medications more accurately, providing new options for high-risk patients through precise drug monitoring and the control of bleeding risk. It also represents the direction of individualized, modern, and precise antithrombotic therapy.

### 3.2. Thromboembolism

Thrombosis constitutes the most common pathological basis of CVD [76]. Occlusion by thrombi at critical sites may trigger fatal outcomes, including ischemic stroke (IS), MI, and pulmonary embolism (PE) [68,77,78,79].Venous thromboembolism, comprising deep vein thrombosis (DVT) and pulmonary embolism, represents the third leading cause of vascular mortality after myocardial infarction and stroke; anticoagulant therapy is the cornerstone of venous thromboembolism management [80].

Patients receiving total hip replacement surgery encounter elevated risks of thromboembolic complications, which is the key disease for evaluating the value of anticoagulant drugs. A randomized double-blind trial compared desirudin with enoxaparin for thromboprophylaxis after primary total hip replacement. An efficacy analysis of 1587 patients showed significantly lower proximal deep vein thrombosis incidence with desirudin compared with enoxaparin, with comparable safety profiles [81]. This demonstrates the potential advantages of desirudin: as a direct thrombin inhibitor, desirudin’s action does not depend on antithrombin III and is not affected by heparin neutralizing factors in the body, thereby potentially providing more stable and predictable anticoagulant effects in the hypercoagulable state after surgery. Although both have comparable safety profiles, desirudin’s significant efficacy advantage favors its application in this field. It also suggests that for the prevention of deep vein thrombosis after total hip arthroplasty, using anticoagulant drugs with a more direct mechanism of action (such as hirudin-like drugs) may be a direction to optimize preventive strategies and break through existing efficacy bottlenecks.

### 3.3. Heparin-Induced Thrombocytopenia (HIT)

Heparin-induced thrombocytopenia (HIT), an immune-mediated adverse drug reaction triggered by heparin-containing anticoagulants, presents as thrombocytopenia with a substantial thrombotic risk, often occurring during the prevention of DVT or post-surgical treatment. HIT-induced thrombotic events (e.g., MI, stroke) directly damage the cardiovascular system and may be life-threatening. The core of treatment lies in immediately discontinuing all heparin and using potent non-heparin anticoagulants. In this clinical situation, recombinant hirudin (such as lepirudin) demonstrates irreplaceable therapeutic value due to its unique mechanism.

A study of 112 HIT patients receiving lepirudin treatment compared 95 eligible patients with historical controls (*n* = 120). Results demonstrated that lepirudin significantly reduced the incidence of mortality, amputation, and new thromboembolic events while maintaining a favorable safety profile in patients with HIT [23]. The fundamental reason is that hirudin, as a direct thrombin inhibitor, can avoid the pathogenic process of HIT immune response: it does not bind to platelet factor 4, thus not inducing or exacerbating platelet activation and procoagulant state. Another clinical trial evaluating recombinant hirudin (lepirudin) for confirmed HIT found 88.7% of acute HIT patients exhibited rapid platelet count increases post-treatment, which effectively prolonged activated partial thromboplastin time (aPTT), demonstrating clinical benefits [82]. This phenomenon indicates that lepirudin effectively breaks the vicious cycle of thrombocytopenia and endothelial injury driven by thrombin by inhibiting excessive thrombin generation, resulting in a controllable prolongation of aPTT, thereby providing patients with crucial antithrombotic effects. Of course, for patients with renal insufficiency, traditional recombinant hirudin (such as lepirudin) is primarily cleared by the kidneys. In patients with renal insufficiency, its half-life is significantly prolonged, increasing the risk of bleeding. Therefore, strict dose adjustment or avoidance is required. As a result, analogs that do not depend on renal clearance (such as bivalirudin) are more advantageous in such patients.

Therefore, hirudin has become a cornerstone of current HIT standard treatment by precisely targeting the core pathological process (excessive thrombin) and completely dissociating from the pathogenic heparin-PF4 complex. It also represents a successful application of the targeted therapy concept based on precise pathological mechanisms in the field of critical illnesses.

## 4. Hirudin Drug Delivery Strategies for CVD

Despite hirudin having multifaceted mechanisms of action against CVD, it has potential adverse effects and clinical risks. A large-scale clinical study indicates that hirudin’s adverse reactions primarily include mild gastrointestinal reactions and allergic responses [73]. Lepirudin, as the first recombinant hirudin in clinical practice, has gradually been replaced by new-generation anticoagulant drugs in first-line clinical drug therapy due to concerns about severe bleeding, immunogenicity issues, and the risk of substitution [67,83].

**Figure 4:**
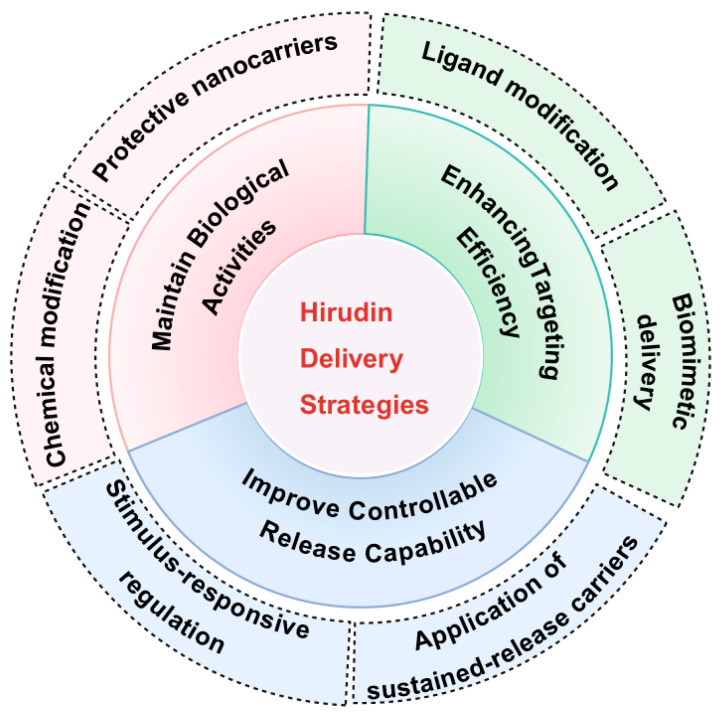
Hirudin drug delivery strategies for CVD.

To overcome these clinical limitations, researchers are actively developing advanced Hirudin nano-delivery strategies to extend the duration of action, reduce dosing frequency, improve patient compliance, and mitigate bleeding risks. The results showed that through polyethylene glycol modification [12,84], liposome wrapping [11,85,86], nanocarrier protection [14,87,88], hydrogel-based sustained release [89,90], or other measures, blood circulation time can be effectively prolonged, bioavailability improved, and targeted delivery of hirudin achieved at local thrombus sites for enhanced therapeutic efficacy. This paper summarizes various treatment strategies for hirudin recently proposed by researchers and discusses the design of hirudin delivery systems from three aspects (Figure 4)—maintaining biological activity, enhancing targeting efficiency, and improving controllable release capability—aiming to contribute to therapeutic efficacy and expand its clinical application value.

### 4.1. Maintain Biological Activity

Hirudin, as a peptide drug, also faces the problems of poor serum stability and susceptibility to proteolytic degradation, which affect its biological activity [89]. Solving the problem of hirudin being easily degraded and inactivated in the body is the basis for designing delivery systems. Currently, chemical modification and the use of protective nanocarriers play a crucial role in addressing the aforementioned problems.

#### 4.1.1. Chemical Modification

Studies have shown that modifying hirudin through covalent conjugation with specific molecules can alter its physicochemical properties and enhance its stability [91].

Polyethylene glycol (PEG), a material widely used for the chemical modification of protein drugs, can enhance the in vivo stability of proteins that are not easily degraded by proteases [92]. Pöschel et al. evaluated polyethylene glycol-conjugated hirudin (PEG-Hirudin) for its effects on antithrombin activity in mortality among maintenance hemodialysis patients [12]; Walenga et al. explored PEG-hirudin in healthy volunteers, demonstrating significant alterations in coagulation parameters and platelet function along with extended plasma half-life and enhanced plasma stability compared with recombinant hirudin [84]. Represented by polyethylene glycol (PEG), this method forms an ‘invisible’ hydration layer on the surface of drug molecules through covalent bonding. This not only effectively shields against protease attacks and extends plasma half-life (e.g., significantly prolonging the half-life of PEG-hirudin) but also reduces immunogenicity. Additionally, Sheffield WP et al. demonstrated that human serum albumin (HSA) has been successfully conjugated with Hirudin variant 3 (HV3), which can effectively slow down the clearance of hirudin, thereby maintaining therapeutic plasma concentrations [93,94]. This strategy has excellent biocompatibility, and its effect of extending the half-life may be superior to PEGylation, while avoiding potential issues with PEG. However, it faces challenges such as the complex structure–activity relationship of fusion proteins and high production and purification costs.

#### 4.1.2. Protective Nanocarriers

Nanocarriers can efficiently load peptide drugs through rational design, providing physical barriers that inhibit enzymatic hydrolysis and chemical degradation, thereby prolonging the half-life and enhancing stability [87]. Nanocarriers not only provide physical protection but also serve as a functionalized platform. The literature reveals the process from simple encapsulation to precise design. Liposomes are composed of an aqueous core surrounded by phospholipid bilayer shells, which can effectively encapsulate hydrophilic hirudin in their core and improve hirudin bioavailability [85]. Positively charged liposomes exhibit greater stability than neutral liposomes due to electrostatic adsorption with negatively charged cells, which enables the slow release of peptides or proteins from liposomes, thereby prolonging their half-life and stability in the bloodstream [86]. Meng et al. confirmed electrostatic binding between the negative charge of hirudin and cationic liposomes. Compared with the neutral liposome group, cationic liposome-associated hirudin enhanced pharmacological efficacy and elevated plasma recombinant hirudin variant-2 (rHV2) concentrations via the parenteral route with good biosafety [11]. This indicates that the fundamental physicochemical properties of the carrier material (such as charge) themselves are a powerful regulatory tool.

Additionally, polymer nanoparticles enhance stability by forming a hydrophobic core, which isolates hirudin from proteases. Cheng et al. used PEG-modified dendritic polymers for hirudin delivery, where PEG shielded the positive charge on the surface of PAMAM, inhibiting rapid clearance by the reticuloendothelial system and prolonging blood circulation time; this is a good example of long-cycle design [14]. Xiao et al. developed a targeted drug delivery platelet-derived nanoplatform (AMSNP@PM-rH/A) loaded with a direct thrombin inhibitor and recombinant hirudin. Targeted DVT treatment was achieved through AMSNP-mediated delivery, which effectively eliminated deep vein thrombosis and enhanced therapeutic efficacy [88]. This study used amination modification of mesoporous silica to improve stability and drug loading performance, applying a step-by-step sequential loading strategy: hydrophobic apixaban dissolved in dichloromethane was embedded into aminated mesoporous silica nanoparticles (AMSNP) via hydrophobic interactions; hydrophilic recombinant hirudin dissolved in physiological saline was adsorbed onto AMSNP surfaces, effectively reducing drug interference and achieving efficient co-loading of hydrophilic and hydrophobic drugs, while providing a highly stable drug core for platelet membrane biomimetic targeting.

These carriers systematically resist enzymatic degradation and rapid renal clearance through steric hindrance effects, laying a foundational platform for subsequent targeted delivery and controlled-release functions.

### 4.2. Enhancing Targeting Efficiency

Hirudin, as a direct thrombin inhibitor, exerts anticoagulant effects by binding to thrombin in the blood [6]; therefore, on the basis of ensuring that the drug has a long circulation, the next step is to guide its accumulation at the thrombus site, which is key to reducing the risk of bleeding; the design of hirudin nano-delivery strategies needs to actively target the thrombus site to reduce systemic distribution while maintaining biological activity.

#### 4.2.1. Ligand Modification

Peptide ligands have become essential tools for targeted drug delivery due to high affinity, low immunogenicity, and good biocompatibility. Han et al. constructed a hirudin prodrug utilizing a dual ligand modification strategy with enhanced anticoagulation precision and safety, achieving targeted delivery to thrombosis sites through specific binding of annexin V to phosphatidylserine on activated platelets; inhibiting thrombin activity using hirudin; and prolonging half-life using albumin binding domain. The collaboration of these three mechanisms achieved efficient thrombus treatment with reduced bleeding risk. This study demonstrates the powerful effectiveness of the multi-functional domain fusion strategy in solving complex biomedical problems [95]. Tian et al. successfully constructed a biomimetic microneedle patch delivery system loaded with a hirudin-based fusion protein prodrug for thrombus targeting and FXa response through genetic engineering technology, where the linker peptide was cleaved explicitly by high-concentration coagulation factor Xa at the thrombus site, achieving on-demand drug release, inhibiting thrombosis, and reducing bleeding risk [96].

#### 4.2.2. Biomimetic Delivery

Biomimetic nanocarriers, which offer superior targeting and excellent biocompatibility due to their natural structures, morphology, size, and surface properties, have garnered significant research interest as carriers for targeted therapeutics [97]. Compared with ligand modification, the limitation lies in its dependence on a single receptor–ligand pair, whereas thrombosis involves multiple molecular interactions. The biomimetic strategy directly replicates cells naturally involved in thrombosis formation. Cell membrane biomimetic nanoparticles, composed of nanoparticles wrapped in cell membranes, utilize membrane proteins to maintain biological functions, prolong circulation time, and achieve targeted delivery [98]. Li et al. developed a platelet membrane-camouflaged nanoplatform based on porphyrin covalent organic frameworks and melanin for targeted delivery of hirudin, leveraging the thrombus-homing properties of platelet membranes. In addition,97% drug loading efficiency, far exceeding conventional carriers [99]. This strategy is based on the fact that platelet membranes themselves are rich in various adhesion receptors (such as GPIbα targeting vWF and GPIIb/IIIa targeting fibrinogen), which can actively target damaged blood vessels and thrombi through multivalent and multi-target interactions. By encapsulating them in nanoparticles, it is similar to ‘disguising’ the nanoparticles as blood cells. This endows the nanoparticles with two major advantages: natural and efficient targeting ability and excellent immune escape capability.

### 4.3. Improve Controllable Release Capability

To enable the drug delivery system to ‘perceive’ disease status and ‘respond’ accordingly, achieving on-demand drug administration. hirudin, as the most potent specific inhibitor of thrombin currently known [14], achieves the controlled release of peptide drugs through material design and environmental response mechanisms using a nano-delivery system, thereby avoiding fluctuations in blood drug concentration and addressing the issues of a short half-life and frequent administration in clinical applications.

#### 4.3.1. Application of Sustained-Release Carriers

Sustained-release carriers are key materials for delivery systems used to control drug release rates and prolong action times [100], which are suitable for the delivery of hirudin. By selecting materials with different degradation characteristics (e.g., PLGA, chitosan), the sustained release of hirudin can be achieved. Research has confirmed that a low-release coating based on vapor-deposited polymer enables the quantitative release of recombinant hirudin from the surface coating within 14 days, achieving sustained release [101]. Wang et al. engineered polyion complex (PIC) micelles using methoxy poly-grafted (ethylene glycol)-chitosan carriers for rHV2. Pharmacokinetic experiments revealed that the micelle carrier significantly prolonged the mean residence time (MRT) of rHV2 in vivo, attributable to PEG-induced ‘invisible’ properties, its nanoscale size, and protective effects of the micelle structure on the drug, which can effectively counteract drug clearance and degradation, enhancing the efficacy of anticoagulant and antithrombotic therapy [14]. Tian et al. developed a biomimetic microneedle patch drug delivery system composed of silk fibroin and chitosan that enabled cfEH to sustain long-term efficacy release for one week. Furthermore, the cfEH/MN patch exhibited better pharmacokinetic behavior and sustained antithrombotic effects, while reducing the risk of bleeding [96]. The aforementioned PIC micelles and microneedle patches can provide a steady, controllable drug concentration over the long term, avoiding peak-trough fluctuations and improving compliance. However, this is an ‘open-loop’ system that cannot respond to acute changes.

#### 4.3.2. Stimulus-Responsive Regulation

Compared with sustained release using controlled-release carriers, stimulus-responsive systems can achieve ‘closed-loop feedback’ and ‘responsive activation’. Previous studies have confirmed that utilizing the thrombotic microenvironment can achieve on-demand drug release in response to high levels of thrombin [102]. Tian et al. designed a long-acting anticoagulant hydrogel based on bivalirudin and microenvironment-responsive activator FXa [103]. After subcutaneous injection, the anticoagulant peptide forms a semi-solid depot resistant to protease degradation. It slowly releases the prodrug into the blood, where it is activated by thrombus-associated protease FXa to inhibit thrombus formation.

This strategy combines target specificity with active control, significantly enhancing the safety of drug administration. This provides a simple strategy for designing supramolecular hydrogels with long-term stability, high drug loading, and on-demand antithrombotic activity [90]. Mo et al. developed thrombin-sensitive nanogels loaded with hirudin, which can self-regulate the hirudin release rate to reduce bleeding risk during anticoagulation. The cross-linking network comprises a thrombin-cleavable peptide (TCP); thrombin specifically cleaves TCP, triggering the disintegration of the nanogel and releasing hirudin variant 3 (HV). The released HV binds to thrombin to form complexes, inhibiting thrombin activity, reducing the TCP cleavage rate, and establishing a closed-loop controlled-release system for intelligent on-demand HV delivery, thereby overcoming the dose-control limitations of traditional anticoagulants [89]. Nagasaki et al. developed a novel pH-sensitive antioxidant nanoparticle loaded with hirudin (hd@iNanoAOX) that was released in acidic environments to maintain the biological activity of hirudin and prolong its half-life [104].

For the long-term administration of anticoagulant therapy, the design of drug delivery systems has evolved from modifying single properties (PEGylation) to constructing multi-functional carriers (nanoparticles), and further, to introducing biological intelligence (targeting, responsiveness). In addition, new routes of administration, such as microneedling, can provide a painless and convenient alternative for hirudin delivery [96,105]. With the advancement of precision medicine, the introduction of 3D printing technology has provided the possibility for personalized hirudin delivery. Three-dimensional printing enables the creation of various microneedle molds, saving time and costs. Wu et al. developed a microneedle array that can adjust parameters according to patients’ specific conditions to achieve tailored, precise drug delivery. The results indicate that MNs loaded with recombinant hirudin and hyaluronic acid (HA) show significant potential for preventing thromboembolic diseases while reducing the risk of bleeding [102]. An ideal system in the future may include a biomimetic-targeting outer shell (platelet membrane), an intelligent responsive core (FXa/thrombin-sensitive gel), and a long-acting sustained-release formulation (microneedle patch) and may combine new technologies (such as 3D printing) to achieve personalized customization.

## 5. Current Challenges and Prospects

Hirudin, a natural anticoagulant peptide, plays a crucial role in CVD through its multi-target regulatory mechanisms, which involve antithrombotic, anti-inflammatory, and anti-myocardial fibrosis effects. Nanotechnology-based delivery systems enhance bioavailability while reducing bleeding risks, thereby addressing limitations such as poor stability and a short half-life. Despite the great potential of these delivery strategies, most new formulations are in the preclinical or early clinical research stages, and they still face challenges in translating from preclinical to clinical settings.

Firstly, the nanocarriers currently used in treatment strategies are challenging to scale up for large-scale production, suffer from low encapsulation rate, and exhibit significant variations between product batches. To overcome these challenges, developing novel carrier materials with high safety and flexible functionalizability is imperative for researchers. Peptides have shown exceptional benefits, including good biocompatibility, degradability, the ability to self-assemble into functional nanostructures, and selective binding to disease targets. Microfluidics-enabled spatiotemporal control of peptide and protein microcarriers provides novel pathways for biomedical applications [106].

Secondly, when used for long-term cardiovascular therapy, it is challenging to achieve regular dose adjustment and the timely delivery of hirudin. Besides conventional sustained-release materials, the system can also incorporate self-feedback mechanisms to utilize microenvironmental cues for real-time release modulation. Due to interindividual variability in response to treatment and the lack of real-time monitoring in clinical practice, advanced monitoring systems may be necessary for personalized regulation. It is usually monitored by activated partial thromboplastin time (aPTT). Intelligent closed-loop systems (CLSs) have promising potential in therapeutic drug monitoring and individualized treatment. Inspired by closed-loop insulin delivery systems, which adjust dosage based on real-time blood glucose monitoring, future AI-powered feedback-enabled smart patches for coagulation biomarker monitoring can be developed to achieve a dynamic assessment of thrombosis risk and self-regulation of hirudin release rates [107].

Finally, it is necessary to establish rigorous in vitro–in vivo correlation standards for thrombus targeting efficiency, drug release kinetics, and other related parameters of the nano-delivery system, following the relevant guidelines issued by regulatory authorities such as the FDA [108]. This evaluation system can be established by leveraging strategies from oncology nanomedicine, combined with microfluidic chips for real-time dynamic assessment of thrombus formation, and utilizing organ-on-a-chip platforms for long-term toxicity study. Notably, some novel technologies developed for oral peptide delivery, such as the SOMA capsule and LUMI microneedle, are currently at the preclinical stage, capable of enhancing the bioavailability of oral insulin and providing novel strategies for overcoming the gastrointestinal challenges associated with hirudin. In addition, the patch–microneedle system holds great promise for clinical application, in expanding the loading capacity and achieving synergistic release; the composite formulation, by integrating the transdermal patch with microneedles, also opens up new paths for the combined therapy of hirudin with other drugs (thrombolytics and antiplatelet drugs) [109].

## 6. Conclusions

Hirudin, as a potent thrombin-inhibiting activity, holds significant value in treating cardiovascular diseases through its antithrombotic, anti-myocardial fibrotic, and anti-inflammatory effects. To address its clinical limitations, nanotechnology-based delivery strategies can effectively enhance the stability, targeting efficiency, and therapeutic durability of hirudin, enabling intelligent on-demand drug release. In the future, by advancing smart delivery systems and integrating novel technologies such as microfluidics and organ-on-a-chip platforms, the precision, long-lasting efficacy, and personalized treatment of hirudin can be achieved. The hirudin delivery system is expected to enable the individualized clinical treatment of CVD, providing patients with safer, more efficient, and personalized therapy.

## Figures and Tables

**Table 1:** Clinical applications of recombinant hirudin and derived formulations.

Name	Molecular Type	Target of Drug Delivery	Half-Life	Indications	Bleeding Risk	Clearance Pathway	Development Status	Reference
Lepirudin (Refludan^®^)	recombinant hirudin	Intravenous infusion	1.3 h	Prevention and treatment of HIT-related thrombosis	High (A narrow therapeutic index with fatal hemorrhage incidence > 5%)	Kidney	Exit the mainstream market	[63,67]
Desirudin (lprivask^®^)	recombinant hirudin	Subcutaneous injection	2–3 h	prevention of DVT after total hip arthroplasty	Medium (Equivalent to enoxaparin)	Kidney	Listed in the EU	[64]
Bivalirudin (Angiomax^®^)	hirudin derivative	Intravenous infusion	25 min	PCI anticoagulation and ACS	Low (Risk reduction of 50% compared with heparin)	Kidney; Protease hydrolysis	Global PCI drug use	[65,66]

## Data Availability

No new data were created or analyzed in this study. Data sharing is not applicable.

## Abbreviations

The following abbreviations are used in this manuscript:

CVD	Cardiovascular diseases
ACS	Acute coronary syndrome
VTE	Venous thromboembolism;
AT-III	Antithrombin III
t-PA	Tissue-type plasminogen activator
rt-PA	Recombinant tissue-type plasminogen activator
ECM	Extracellular matrix
PAR-1	Protease-activated receptor 1
CaN	Calcineurin
Ang II	Angiotensin II
AT1R	Angiotensin II receptor 1
SHR	Spontaneously hypertensive rat
TGF-β	Transforming growth factor-β
MMP-2	Matrix metalloproteinase-2
MMP-9	Matrix metalloproteinase-9
COL-I	Type I collagen
COL-III	Type III collagen
MAPK	Mitogen-activated protein kinase
MMP-1	Marker matrix metalloproteinase-1
TIMP-2	Tissue inhibitor of metalloproteinase-2
DAMPs	Damage-associated molecular patterns
TLRs	Toll-like receptors
PARs	Proteinase activated receptors
HUVECs	Human umbilical vein endothelial cells
HIT	Heparin-induced thrombocytopenia
STEMI	ST-segment elevation myocardial infarction
NSTEMI	Non-ST-segment elevation myocardial infarction
UA	Unstable angina
MI	Myocardial infarction
PCI	Percutaneous coronary intervention
IS	Ischemic stroke
PE	Pulmonary embolism
DVT	Deep vein thrombosis
aPTT	Activated partial thromboplastin time
PEG	Polyethylene glycol
HAS	Human serum albumin
RES	Reticuloendothelial system
DTI	Direct thrombin inhibitor
AMSNP	Aminated mesoporous silica nanoparticles
PS	Phosphatidylserine
ABD	Albumin binding domain
rHV2	Recombinant hirudin variant-2
MRT	Mean residence time
TCP	Thrombin-cleavable peptide
HV	Hirudin variant 3
CLSs	Closed-loop systems
